# A Review of the Hematological Picture of Severe COVID-19 Infection

**DOI:** 10.7759/cureus.78797

**Published:** 2025-02-09

**Authors:** Dominga Amoroso, Stefania Bongo, Anna Copponi, Vanessa Rossi, Roberta Di Giorgio, Sergio Bernardini, Lorenzo Ippoliti, Maria Morello

**Affiliations:** 1 Department of Experimental Medicine, Faculty of Medicine, University of Rome Tor Vergata, Rome, ITA; 2 Department of Biomedicine and Prevention, University of Rome Tor Vergata, Rome, ITA

**Keywords:** blood count, blood film microscopy, covid-19, hematological parameters, morphological abnormalities

## Abstract

Numerous hematological abnormalities have been documented in COVID-19 patients. We conducted an analysis of 82 articles from PubMed, focusing on the hematological characteristics observed in survivors (S) and non-survivors (NS) with moderate and severe COVID-19 symptoms, respectively. Our review underlines neutrophilia, lymphopenia, and thrombocytopenia as hallmark features of the disease. In severe cases, blood cell microscopy revealed the following abnormalities: i) an increased number of neutrophils, often displaying granularity, toxic granulation, and vacuolization; ii) lymphocytes with a notably blue cytoplasm; iii) several monocytes that contain vacuoles; iv) platelet aggregation; and v) basophilic stippling in red blood cells. Furthermore, scattergram analysis of COVID-19 patients revealed two common features: i) an increased neutrophil population and ii) the presence of a distinctive "sandglass pattern". This review underscores the critical role of hematochemical and cytomorphological blood cell analysis in COVID-19 patients, aiding clinicians in better recognizing and understanding the indicators of disease severity.

## Introduction and background

In early 2020, the world was thrust into a global health crisis with the onset of COVID-19, which posed a severe challenge to the resilience and capacity of healthcare systems worldwide. SARS-CoV-2, the virus responsible for the disease, leads to a highly variable range of symptoms, from mild to life-threatening, underscoring the unpredictable nature of the pandemic. Understanding the pathophysiology of the disease is essential for effective crisis management, and in this context, the laboratory plays a critical role in providing the clinically relevant information needed to diagnose and monitor SARS-CoV-2 infection. Among the key diagnostic tools, the complete blood count (CBC), also known as the “hemogram,” has proven to be crucial [1-31]. The CBC is essential for diagnosing and monitoring a wide range of conditions, including anemia, infections, inflammation, coagulation disorders, hematologic malignancies, and, in particular, COVID-19 [14,16]. An important aspect of CBC analysis is the examination of white blood cell (WBC) scattergrams, which reveal crucial information to assess the severity of COVID-19 infection [12-17]. Since patients with COVID-19 frequently present significant hematological changes, a detailed analysis of blood cell morphological alterations can provide crucial information into the disease course [1,17,19]. However, despite numerous studies exploring the use of the CBC as a diagnostic tool for COVID-19, few systematic reviews that summarize the complete picture of the main hematological and morphological indicators typical of COVID-19 exist in the literature to date. This review aims to fill these gaps in the literature. The goal is to provide a clearer and more systematic overview that, in addition to promoting a more complete understanding of the disease progression, can be of practical use to laboratory scientists and clinicians. In particular, this review provides a comprehensive view of the patient with severe COVID-19, allowing laboratory professionals to focus on critical instrumental and morphological parameters during reporting. This will help improve the ability to timely monitor disease severity and support more targeted and timely therapeutic decisions.

## Review

**Figure 1:**
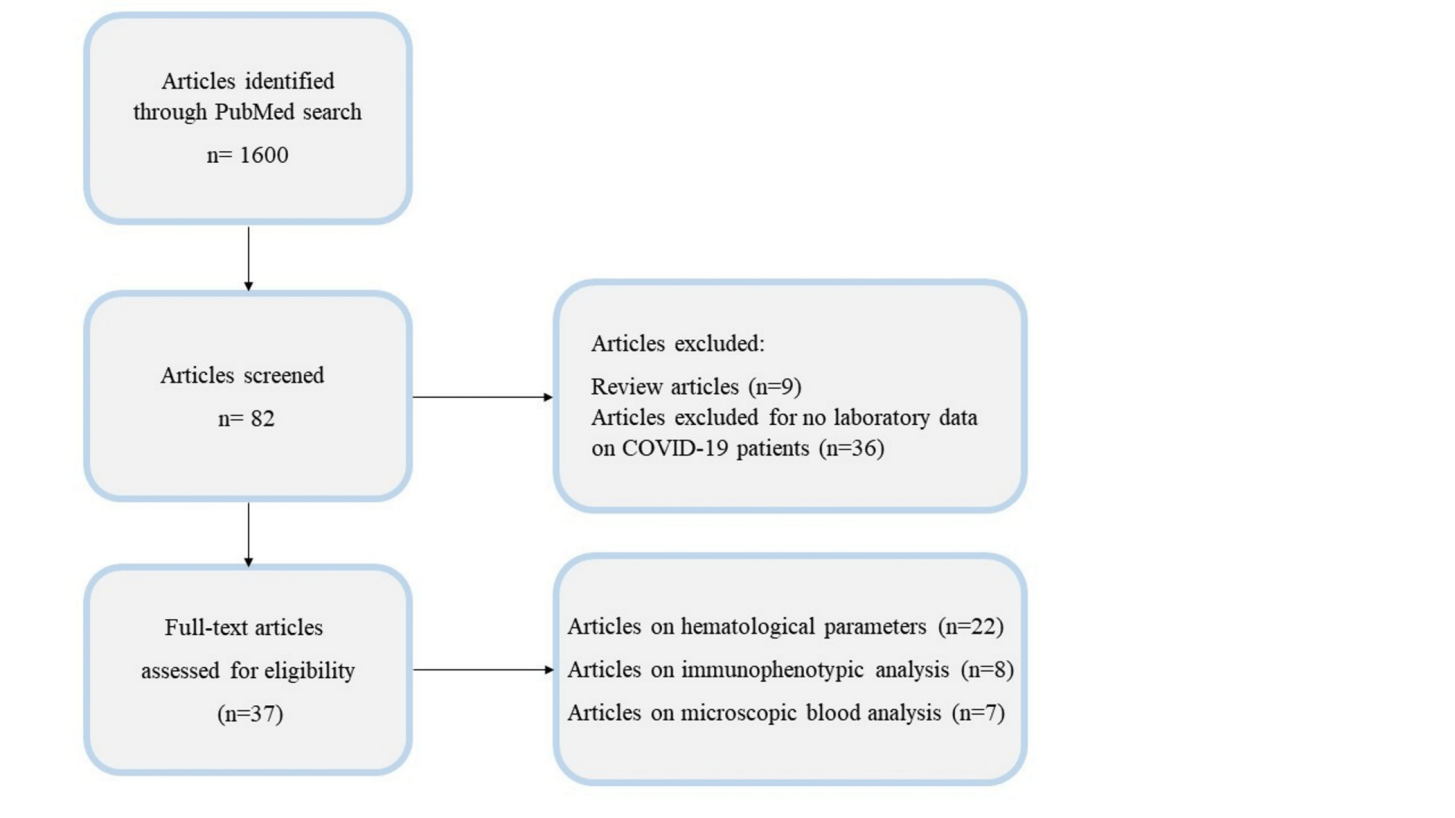
Workflow for screening relevant COVID-19 articles.

We conducted this study by searching PubMed, the most recent articles published between February 2020 and January 2024 using the keywords: "COVID-19," "hematological parameters," "morphological abnormalities”, and "blood count." From a total of 1600 articles obtained by the initial literature search, we identified 82 articles. However, we excluded nine review articles and 36 research articles that did not provide detailed information on CBC analysis. The primary objective of our study was to support clinicians in identifying and understanding the effects of SARS-CoV-2 on blood cells in survivors as well as in patients with moderate and severe forms of the disease. Therefore, we focused on reviewing the main alterations in hematological parameters, specifically: i) scattergram analysis and ii) morphological changes in blood cells. Ultimately, we included 37 articles in this review, which described the hematological characteristics of 2249 survivors (S), 1152 non-survivors (NS), 3599 cases with moderate COVID-19 infections, and 2144 severe cases. In detail, of this total, in three articles [1-3], S and NS patients were extrapolated from both moderate and severe groups, instead in four articles [4-7], they were obtained by considering only severe group of patients. Of these 37 studies, i) 22 examined hematological parameters, ii) eight reported on immunophenotypic analysis, and iii) seven analyzed blood cells at the microscopic level (see Figure 1). 

Alteration of hematological parameters in COVID-19

**Figure 2:**
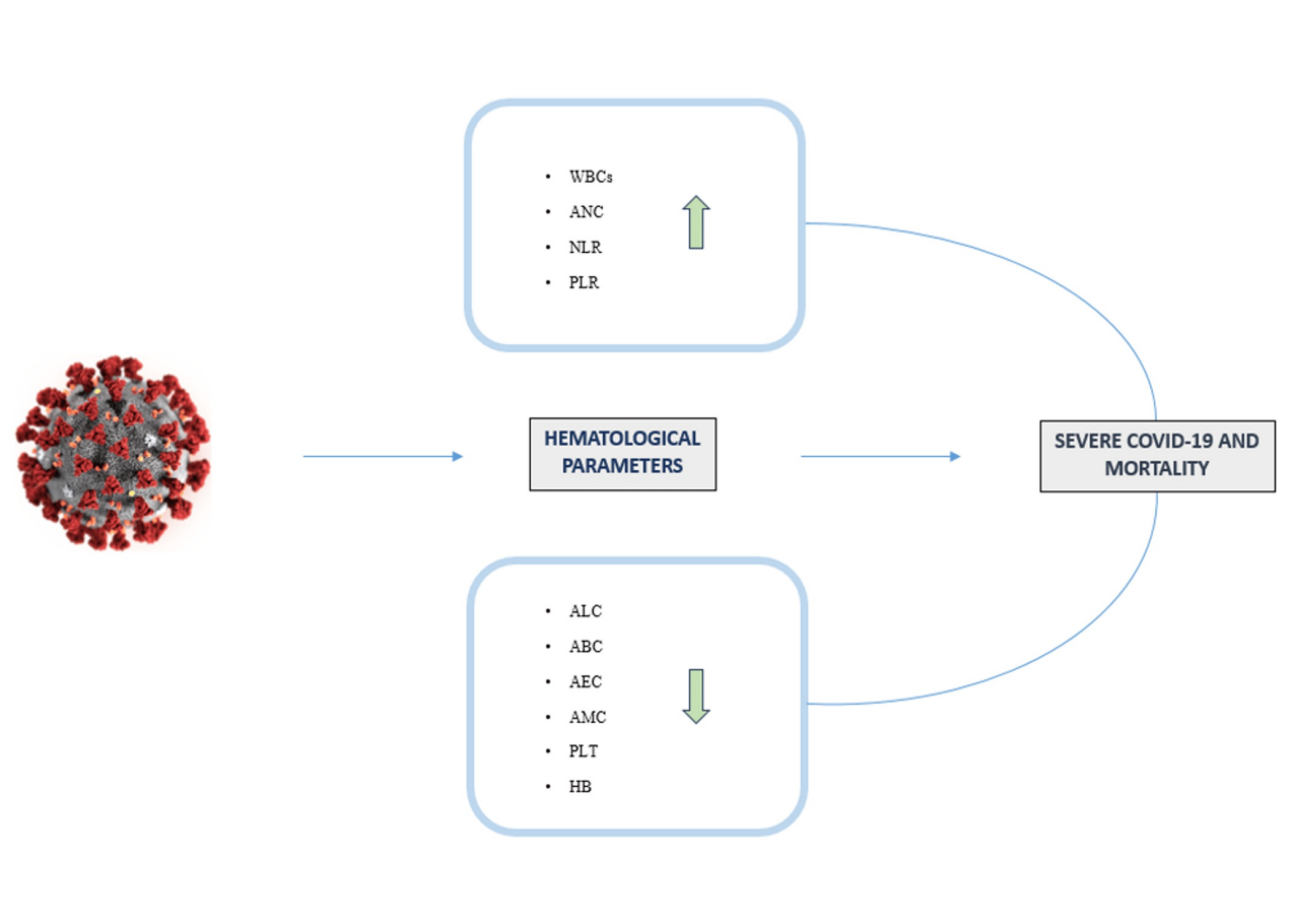
Main alterations of hematological parameters and their correlation with disease outcomes. WBCs, White blood cells; ANC, Absolute neutrophil count; NLR, Neutrophil lymphocyte ratio; PLR, Platelet lymphocyte ratio; ALC, Absolute lymphocyte count; ABC, Absolute basophil count; AEC, Absolute eosinophil count; AMC, Absolute monocyte count; PLT, Platelet count; Hb, Hemoglobin. The figure was created by the authors using Biorender (https://www.biorender.com/).

In patients with severe COVID-19 symptoms, distinctive hematological changes have been reported across multiple studies [8-14]. As shown in Figure 2, WBC analysis frequently reveals leukocytosis (defined by an absolute WBC count > 11 x 10^9/L). This elevation is primarily driven by an increase of neutrophils with counts surpassing 7.7 x 10^9/L. Given that hyperinflammation is characterized by a cytokine storm which is one of the principal pathogenic mechanisms of COVID-19, the rise of neutrophils and their degranulation are well documented in the literature. In fact, numerous studies have reported elevated WBC and neutrophil counts in patients with a severe form of COVID [1,2,4,5,15-22], and particularly in NS patients [1-3,5,6,23-28]. 

Additionally, lymphopenia defined by an absolute lymphocyte count below <1.0 x 10^9/L is commonly observed in COVID-19 patients. This reduction seems due to the expression of the ACE2 receptors on the surface of lymphocytes and the infiltration of these cells into inflamed lung tissue [19]. Some studies suggest that the lymphocyte depletion observed in the severe form of COVID-19 infection could be linked to the loss of immune cells, including CD4+ and CD8+ T cells (with CD8+ cells playing a critical role in destroying virus-infected cells), B cells, and natural killer (NK) cells [7,29,30] (see Table 1).

**Table 1:** Summary of the hematological profile of moderate (M), severe (SE), survivor (S), and non-survivor (NS) COVID-19 patients NLR: Neutrophil-to-lymphocyte ratio

Authors	Hemoglobin g/dl				Platelets x 10^3^/μl				WBCs x 10^3^/μl				Neutrophils x 10^3^/μl				Lymphocytes x 10^3^/μl				Basophils x 10^3^/μl				Eosinophils x 10^3^/μl				Monocytes x 10^3^/μl				NLR				PLR			
	M	SE	S	NS	M	SE	S	NS	M	SE	S	NS	M	SE	S	NS	M	SE	S	NS	M	SE	S	NS	M	SE	S	NS	M	SE	S	NS	M	SE	S	NS	M	SE	S	NS
Awoke et al., 2023 [1]	13.5	13.3	13.6	12.7	191.5	181	193	172	6.3	7.9	6.4	9	4.7	6.8	4.9	7.9	0.8	0.7	0.9	0.6	-	-	-	-	-	-	-	-	-	-	-	-	4.78	10.41	4.8	13.8	200.4	242.6	193.6	301.9
Khatib et al., 2023 [2]	12.1	11.5	11.9	11.8	259	212	252	240	14	21	11	16	10	28	8.1	22.5	1.2	0.6	1.6	0.7	-	-	-	-	-	-	-	-	-	-	-	-	9.5	52.5	6.7	42.6	0.03	0.05	197.2	555
Urbano et al., 2022 [3]	12	12.6	12.4	11.2	211	184	221	184	6.8	6.6	6.2	8.9	3.6	1.6	3.4	4.9	1.7	2.4	1.7	1.6	-	-	-	-	-	-	-	-	-	-	-	-	-	-	-	-	120.6	59.4	122.9	94.5
Awale et al., 2022 [4]	11.7	11	-	-	164	155	-	-	6.75	11.9	-	-	4.6	9.7	-	-	1.2	0.7	-	-	-	-	-	-	-	-	-	-	0.4	0.3	-	-	3.7	18	-	-	-	-	-	-
Ben Jemaa et al., 2022 [5]	13	12.4	12.4	12.5	233	254	253	255	9.1	11.6	8.3	12.5	7.04	9.63	6.8	10.4	1.41	1.16	0.93	1.22	0.012	0.045	0.008	0.053	0.048	0.083	0.055	0.089	0.69	0.5	0.49	0.5	6.84	12.96	-	-	-	-	-	-
Tong et al., 2021 [6]	-	-	12.5	10.7	-	-	232	145	-	-	5.42	6.73	-	-	3.36	5.58	-	-	1.08	0.50	-	-	0.01	0.00	-	-	0.03	0.00	-	-	0.47	0.33	-	-	3.05	8.43	-	-	-	-
Mahmood et al., 2023 [16]	11.23	12.03	-	-	246	227	-	-	17	22	-	-	15	15	-	-	5	6	-	-	-	-	-	-	-	-	-	-	-	-	-	-	2.70	6.87	-	-	131.5	238.3	-	-
Abdulla et al., 2022 [17]	12.8	12.2	-	-	-	-	-	-	7.86	9.70	-	-	6.78	7.49	-	-	4.85	2.27	-	-	0.54	1.02	-	-	1.23	0.47	-	-	-	-	-	-	2.99	11.56	-	-	-	-	-	-
Attia et al., 2023 [18]	13.1	12.5	-	-	214	180	-	-	8.9	10.5	-	-	6.9	8.5	-	-	1.2	0.8	-	-	-	-	-	-	0	0	-	-	2.9	1.1	-	-	4.3	10.6	-	-	-	-	-	-
Waris et al., 2021 [19]	12.9	13.1	-	-	224	165	-	-	6.76	11.79	-	-	4.4	8.8	-	-	1.9	1.4	-	-	-	-	-	-	-	-	-	-	-	-	-	-	2.70	6.87	-	-	131.5	238.3	-	-
Qin et al., 2020 [20]	-	-	-	-	-	-	-	-	4.9	5.6	-	-	3.2	4.3	-	-	1.0	0.8	-	-	0.0	0.0	-	-	0.0	0.0	-	-	0.4	0.4	-	-	3.2	5.5	-	-	-	-	-	-
Liao et al., 2020 [21]	12.2	11.5	-	-	198	105	-	-	5.05	9.33	-	-	3.22	8.08	-	-	1.2	0.54	-	-	0.01	0.03	-	-	0.04	0.01	-	-	0.46	0.38	-	-	2.67	16.02	-	-	-	-	-	-
Eijmael et al., 2021 [22]	-	-	-	-	-	-	-	-	5.2	7.8	-	-	3.6	5.8	-	-	0.9	0.9	-	-	0.01	0.01	-	-	0.01	0.00	-	-	0.5	0.5	-	-	-	-	-	-	-	-	-	-
Deng et al., 2020 [23]	-	-	-	-	-	-	-	-	-	-	4.52	7.23	-	-	-	-	-	-	1	0.63	-	-	-	-	-	-	-	-	-	-	-	-	-	-	-	-	-	-	-	-
Pertiwi et al., 2023 [24]	-	-	-	-	-	-	354	271	-	-	8.8	12.3	-	-	5.9	10	-	-	1.94	1,07	-	-	-	-	-	-	-	-	-	-	-	-	-	-	4.6	11.7	-	-	224.7	321.6
Bellan et al., 2020 [25]	-	-	13.9	13.3	-	-	197	159	-	-	6.3	7.5	-	-	4.5	6.4	-	-	1.2	0.9	-	-	-	-	-	-	-	-	-	-	-	-	-	-	-	-	-	-	-	-
Paliogiannis et al., 2020 [26]	-	-	12.8	12.8	-	-	210	161	-	-	5.7	11.6	-	-	3.70	10.05	-	-	1.00	0.60	-	-	-	-	-	-	-	-	-	-	0.31	0.30	-	-	3.7	12.2	-	-	191	227
Khadzhieva et al., 2023 [27]	-	-	-	-	-	-	-	-	-	-	-	-	-	-	-	-	1.24	0.77	-	-	-	-	-	-	0.02	0.01	-	-	-	-	-	-	-	-	-	-	-	-	-	-
Ortega-Rojas et al., 2022 [28]	-	-	13.7	13.3	-	-	276	259	-	-	8.7	12.8	-	-	7.4	11.7	-	-	0.9	0.8	-	-	-	-	-	-	-	-	-	-	-	-	-	-	8.6	14.8	-	-	28.2	43.7
Yang et al., 2020 [29]	-	-	12.7	12.9	-	-	164	191	-	-	-	-	-	-	-	-	-	-	-	-	-	-	-	-	-	-	-	-	-	-	-	-	-	-	-	-	-	-	-	-
Guan et al., 2020 [31]	13.5	12.8	-	-	172	137	-	-	4.9	3.7	-	-	-	-	-	-	1	0.8	-	-	-	-	-	-	-	-	-	-	-	-	-	-	-	-	-	-	-	-	-	-
Mu et al., 2021 [32]	-	-	-	-	-	-	-	-	-	-	-	-	-	-	-	-	1.24	0.77	-	-	-	-	-	-	0.02	0.01	-	-	-	-	-	-	-	-	-	-	-	-	-	-

In COVID-19 patients, a significant increase of neutrophil, combined with a concurrent decrease of lymphocyte count, expressed as the neutrophil-to-lymphocyte ratio (NLR), has emerged as a critical inflammatory marker particularly useful in assessing the progression of disease severity diseases [5]. An NLR value below 3 is considered normal, while a value above 3 indicates an acute infection. Notably, elevated NLR levels are observed in severe cases of COVID-19 [1,2,4,5,16-21,31,32]. Furthermore, a low lymphocyte count is associated with a higher risk of mortality [1,2,6,23-27]. Several studies have also highlighted that increased NLR levels are particularly pronounced in NS patients confirming a direct correlation between high NLR and mortality [1,2,6,24,26, 28,33]. In addition to the NLR, other inflammatory indices, such as the platelet-to lymphocyte ratio (PLR) and monocyte-to lymphocyte ratio (MLR), have been evaluated in numerous studies. The normal reference range for PLR is typically around 50-150 [19], and in COVID-19 patients, an elevated PLR has been associated with longer hospital stay and a poorer worse prognosis [1,2,15,19,24,26,28]. These findings emphasize the importance of monitoring both NLR and PLR values that are useful for early identification of critically COVID-19 patients (see Table 1). 

Additionally, the measurement of basophil and eosinophil counts is crucial for evaluating the severity of COVID-19 infection. These cells play a key role in enhancing immunological memory responses and maintaining immune homeostasis. A decrease of basophils and eosinophils has been linked to the severity of COVID-19. Specifically, a reduction in basophils (normal concentration: 0 - 0.2 x 10^9/L) and eosinophils (normal circulating value: 0.02 - 0.5 x 10^9/L) may result from mechanisms such as viral encapsulation in basophils and the infiltration of eosinophils into inflammatory sites or the suppression of eosinophil production in the bone marrow (eosinophilopoiesis) [13].

Extensive research studies have demonstrated that both basophil count and eosinophil count decrease in patients with severe COVID-19, with this reduction being particularly pronounced in NS patients [6,20]. Furthermore, several studies have highlighted that a marked eosinophilopenia was observed in severe cases [21,22,32]. However, contrasting findings have been reported; for instance, Ben Jemaa et al. observed an increase in basophil and eosinophil count in severe COVID-19 cases. This discrepancy may be attributed to differences in race and ethnicity among patients [5].

Monocytes, which play a crucial role in maintaining cellular homeostasis, particularly during inflammation and infections, have also been studied in COVID-19 patients [34]. In severe cases of COVID-19, patients often exhibit a reduction in the monocyte count [4,5,18]. Monocytes and macrophages are essential cells of innate immunity contributing to the inflammatory response by producing pro-inflammatory cytokines and by processing and presenting antigens to T lymphocytes. Their role in the pathogenesis of COVID-19 has been highlighted by the presence of activated tissue macrophages in the lungs of NS patients [18,35].

The viral infections in COVID-19 significantly impact the hematopoietic system leading to a reduction of platelet count. Monitoring platelet levels is critical in infectious diseases, and in COVID-19, thrombocytopenia may arise from several mechanisms: i) a direct viral interaction with bone marrow cells inhibiting platelet production, ii) lung damage resulting in platelet aggregation promoting micro-thrombi formation in the lungs and a consequent platelet consumption likely due to the destruction of megakaryocytes progenitor cells and iii) platelet disruption due to an autoimmune response [13].

**Figure 3:**
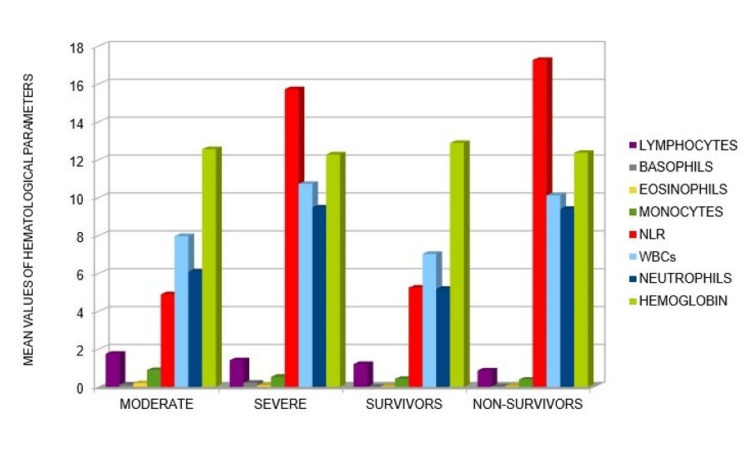
Graphic representation of mean values of hematological parameters detected in COVID-19 patients with different outcomes. This representation was obtained from the analysis of the articles reported in Table 1. In particular, an increase of WBC, ANC and NLR was seen to correlate with worse outcomes of disease (especially in NS COVID-19 patients).

Thrombocytopenia is a distinctive feature commonly observed in severe cases of COVID-19 and it is associated with poorer outcomes [1,3,6,18,19,21, 24-26,28]. Hemoglobin levels also serve as an indicator of disease progression, with lower levels detected in both S and NS of COVID-19 patients [1,3,6,25] (see Table 1). In our analysis of 22 articles, we compiled a table (Table 1) containing the hematological parameters reported in COVID-19 articles. From the data reported in Table 1, we have detected that the key hematological parameters that increase in severity and among cases of NS patients are NLR, white blood cell count, and neutrophil count (see Figure 3).

Immunophenotypic changes in COVID-19

COVID-19 infection disrupts the balance between innate and adaptive immunity, a disruption that is particularly pronounced in severe cases. The acute phase of infection is characterized by marked lymphopenia and significant impairment of both inflammatory and immune responses. The measure of the number of lymphocytes, specifically T cells, B cells and NK cells, is crucial for assessing the extent of immune system alteration [36,37]. An elevated CD4/CD8 ratio is indicative of a cytokine storm trigger, underscoring the severity of the aberrant immune response. NK cells play a vital role in viral elimination by production of cytokines and the secretion of granules containing enzymes such as perforin and granzyme [38]. CD8+ T cells are highly effective at eliminating the virus through their cytotoxic effect, while CD4+ T cells (helper T lymphocytes) that regulate the function of both CD8+ cells and B cells are involved in the clearance of viral infection [30,38]. 

A review of eight articles on immunophenotypic cells analysis revealed notable findings in COVID-19 patients. In 481 patients affected by moderate form and 505 with severe cases, a decrease in T cells (both CD4+ T cells and CD8+ cells) was observed [7,30,39-46]. Additionally, reductions in B cells [7,30,40,41,43,44] and NK cells [30,41-44] were noted as characteristic effects of the infection. An increase of CD4/CD8 was reported in severe cases [39,41,42], although some studies did not find any significant differences [7,40,43,44] (see Table 2). 

**Table 2:** Analysis of the lymphocyte immunophenotype in COVID-19 patients. Notably, there is a decrease in CD4+ and CD8+ lymphocyte subpopulations (↓) and an increase in the CD4/CD8 ratio (↑).

T-Cell Subsets	COVID-19
CD4+	↓ Yang et al., 2021 [39]; Wang et al., 2020 [40]; Jiang et al., 2020 [42]; Liu et al., 2020 [43]; Kazancioglu et al., 2021 [44]; Chen et al., 2020 [45]; Liu et al., 2021 [46]
CD8+	↓ Yang et al., 2021 [39]; Wang et al., 2020 [40]; Jiang et al., 2020 [42]; Liu et al., 2020 [43]; Kazancioglu et al., 2021 [44]; Chen et al., 2020 [45]; Liu et al., 2021 [46]
CD4+/CD8+	↑ Yang et al., 2021 [39]; Jiang et al., 2020 [42]

As highlighted in several compelling articles, the reduction in T cell subsets observed in severe COVID-19 patients does not seem to result from direct viral infection, as T cells lack ACE2, the receptor for SARS-CoV-2. Instead, ACE2 expression on macrophages in secondary lymphoid organs suggests that infected macrophages may trigger lymphocyte apoptosis, potentially through the upregulation of FAS expression [47] Additionally, viral infected macrophages could contribute to lymphocyte apoptosis by releasing of pro-inflammatory cytokines such as IL-2, IL-6 and TNF-α. Given that lymphopenia is a prominent feature in COVID, it possible that this condition results from lymphocyte sequestration in various organs, including lungs, gastrointestinal tract and lymphoid tissues [47-49].

In COVID-19 patients, particularly in NS, an uncontrolled activation of monocytes and an overproduction of inflammatory cytokines (known as a cytokine storm) have been reported, leading to significant tissue damage. Blood analysis has shown that inflammatory monocyte subpopulations, specifically CD14+ and CD16+, are predominant in NS patients, contributing to heightened inflammatory activity [34,50,51]. These findings suggest that enhancing cellular immunity (or regulate its action) could be a key strategy in the treatment against the cellular effect of COVID infections.

Peripheral blood smear prognostic role in COVID-19

**Figure 4:**
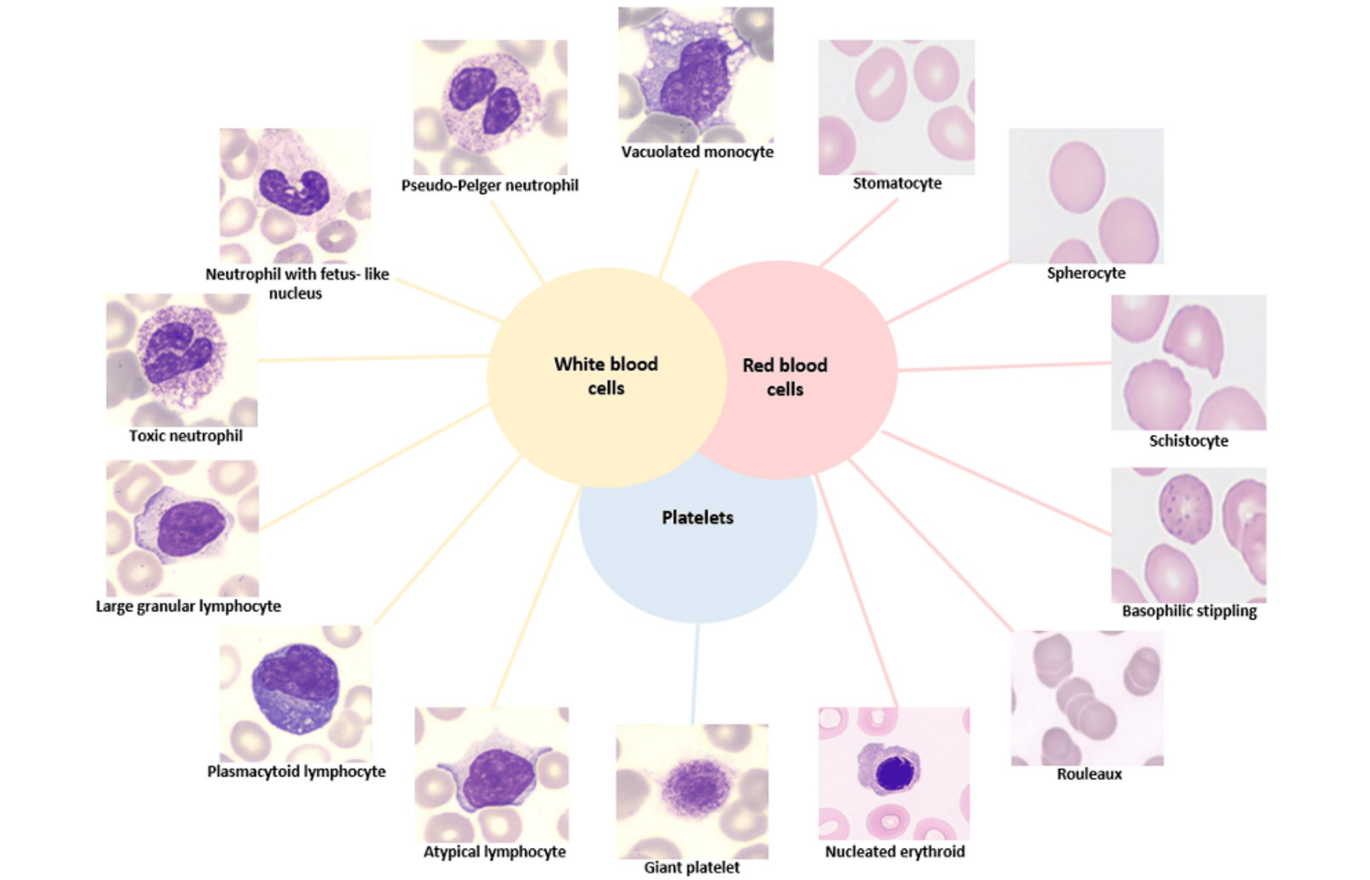
Main morphological alterations of circulating cells from peripheral blood smear in the severe form of COVID-19. Stain: May-Grunwald-Giemsa, (original magnification X100). The figure was created by the authors using images from Hematology Laboratory of Tor Vergata University Hospital.

During COVID-19 infection, the inflammatory response and the alteration of the hematopoietic system lead to significant morphological changes of peripheral blood cells [52,53]. To accurately understand the pathogenicity of COVID-19 and make precise prognostic assessments, a detailed morphological evaluation of blood cells by a peripheral blood smear (PBS) is essential. Microscopic analysis of blood smears allows the evaluation of various alterations, including changes in the size and shape of erythrocytes, as well the evaluation about the morphology of leukocytes and platelets. In this study, we have aimed to provide a comprehensive review and schematic representation of common morphological changes observed in PBSs, especially in the severe form of COVID-19 (see Figure 4). 

Microscopic analysis of WBCs

As reported in several microscopical analyses, the blood from patients affected by the severe form of COVID-19 has revealed several distinctive features [54-60]. Notably, these patients exhibit activated lymphocytes with aberrant characteristics. A PBS shows the presence of reactive or “plasmacytoid lymphocytes” which are characterized by dark blue cytoplasm, an eccentric or indented nucleus with dense chromatin, and a perinuclear Golgi apparatus [18,57-59,61-63]. Additionally, some large granular lymphocytes characterized by abundant cytoplasm and containing distinctive azurophilic granules have been reported [18,54,57-59,64]. In the early phase of COVID-19 infection, a common morphological alteration observed is the particular “left-shift” in myeloid series that is indicative of the presence of immature promyelocytes and metamyelocytes [18,54,57-59,62,65]. In PBS, smudged neutrophils and neutrophils exhibiting toxic features, such as toxic granulation and cytoplasmic vacuolation, are also frequently described [54]. Specifically, these neutrophils may exhibit dark, enlarged, and basophilic granules, which are irregularly distributed and represent the primary (azurophilic) granules of myeloid precursors. These azurophilic granules contain enzymes such as hydrolase, myeloperoxidase, and peroxidase. These features are likely due to cytokine-driven stimulation of myelopoiesis, leading to increased production of lysosomal enzymes [18,58-60,64,65]. The presence of abnormal nuclear shapes such as fetal like-nuclei [58,64] and dysmorphic cells with a complete absence of nuclear lobation, resembling pseudo‐Pelger-Huët morphology, have been frequently described in studies of COVID-19 blood smears [55,57-60,65-67]. These morphological changes are often linked to mutations in the lamin B receptor (LBR), a protein important for nuclear assembly the binding of nuclear membrane to heterochromatin. Pelger-Huët cells are characterized by a reduced nucleus-to cytoplasm ratio, coarsely clumped nuclear chromatin, and unequal lobes with a “dumbbell” or “pince-nez” appearance. These morphological features are commonly associated with various hematologic disorders, including polycythemia vera, multiple myeloma, megaloblastic anemia, and plasmacytoma. They are also linked to non-hematologic disorders such as lupus erythematosus, malaria, influenza, tuberculosis, and HIV. Such abnormalities often reflect a “reactive condition” which is also characteristic of severe infections like the severe form of COVID-19 [68]. Additionally, other studies have observed in smear from COVID patients also the presence of apoptotic cells with granulated nuclear chromatin [64,65]. Regarding the main morphological changes of monocytes, the presence of cytoplasmic vacuoles has been reported [18,59,60,62,64,67]. 

Microscopic analysis of RBCs and platelets

Various abnormalities in RBCs have been reported in COVID-19 patients, indicating significant impacts on RBC physiology. Observed anomalies include polychromasia [65], anisocytosis [54,69], stomatocytes [63,69], spherocytes [63,69], and schistocytes [54,69]. Schistocytes are particularly noted in patients with microangiopathy and vascular thrombosis [70]. Marchi and colleagues [69] identified speculated cells as a common RBC anomaly in COVID-19, with features that are intermediate between echinocytes and acanthocytes. These speculated cells are thought to result from alterations in the lipid or protein composition of the RBC membrane, which can reduce the deformability of the cells and impair their oxygen transport capabilities [71]. Other notable findings include the presence of nucleated erythrocytes [54,62,65], rouleaux formation [65] and basophilic stippling [58,62,65] in peripheral blood smears from severe COVID-19 patients.

Nucleated erythroids can be indicative of various pathological condition including regenerative anemia, lead poisoning, bone marrow disease, hemangiosarcoma, septicemia, and endotoxic shock. Rouleaux formation, where RBCs stack together in aggregates can occur due to high fibrinogen levels or reduced surface charge on the RBCs, which may be associated with conditions such as rheumatoid arthritis and multiple myeloma, as well as transiently during inflammation or acute infections like COVID-19 [72]. Basophilic stippling, characterized by small basophilic inclusions in the RBC cytoplasm formed by RNA, is observed in various conditions including thalassemia, hemolytic anemias, and unstable hemoglobinopathies. There are hypotheses suggesting that such abnormalities may also occur in the RBCs of COVID-19 patients [73].

In addition, platelet morphology in COVID-19 patients frequently shows significant abnormalities, including giant platelets of varying sizes [18,54,55,58,59,62,64,66,67] and platelet clumping. These morphological changes are detected through optical microscopy. Luke et al. confirm these abnormalities using electron microscopy [74], while Kondratov and colleagues also validate pathological erythrocyte forms with electron microscopy [75]. Given that identifying morphological alterations in blood cells is a routine laboratory test, an accurate microscopic analysis of peripheral blood is crucial in COVID-19. This analysis aids clinicians in assessing disease severity and helps in better understanding and elucidating the complex pathophysiology of the infection (see Figure 4).

WBC scattergram in the evaluation of COVID-19 infection 

**Figure 5:**
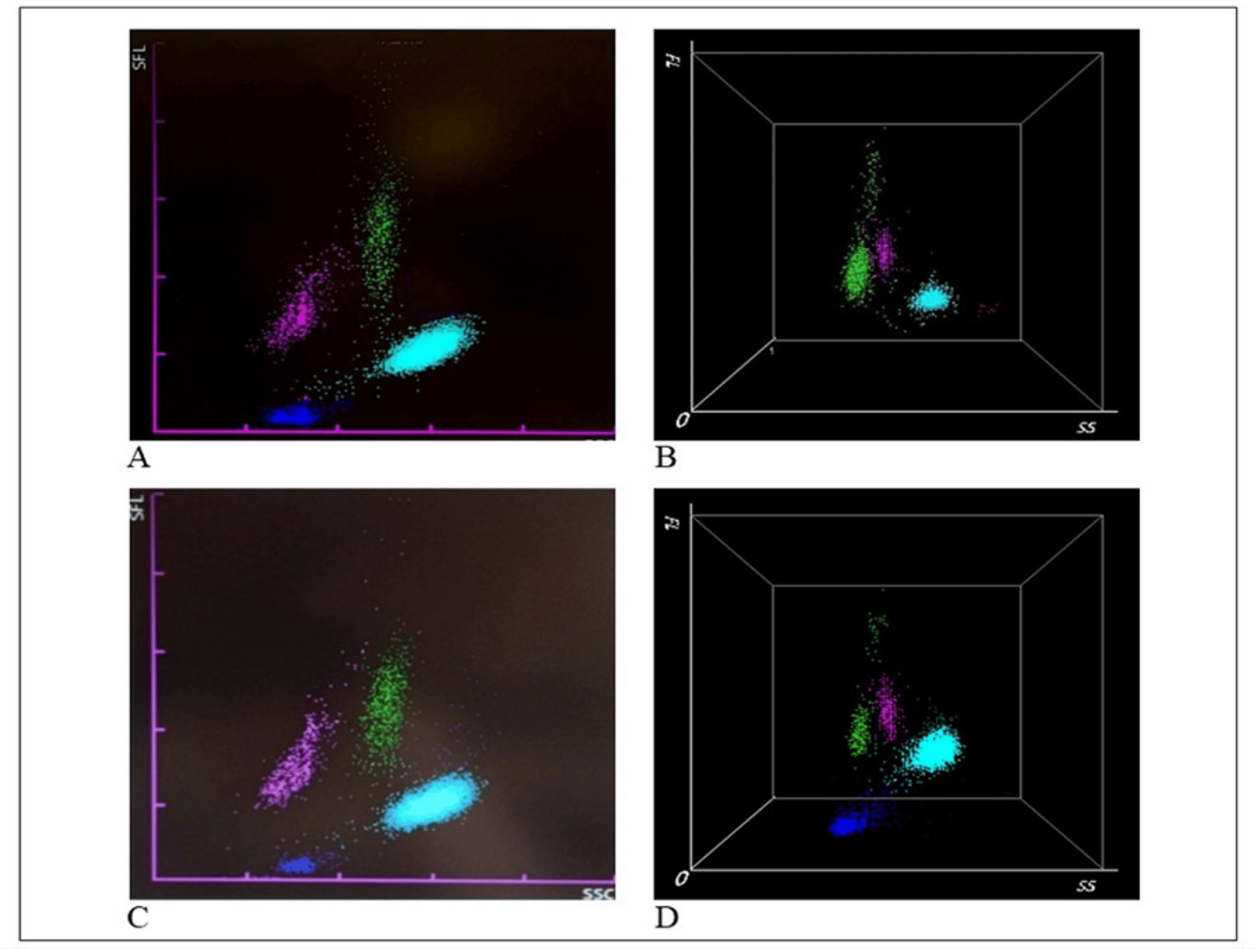
Examples of WDF scattergrams generated by the Sysmex and Mindray hematology analyzers. Sysmex XN-9000 (A, C) and Mindray BC-6800 Plus (B, D) hematology analyzers. The scattergrams from COVID-19 patients exhibit atypical white blood cell (WBC) patterns, including neutrophilia (NE, in light blue), lymphopenia (LY, in pink), and monocytes (MO, in green). A characteristic "sandglass" effect is visible in the green spots on scattergrams B and D. The figure was created by the authors using images from Hematology Laboratory of Tor Vergata University Hospital. WDF, White blood cell differential fluorescence

Hematology analyzers (HAs) are essential tools in clinical laboratories useful for performing CBC tests and providing accurate and reproducible results. HAs are largely used to count and to differentiate nucleated cells yielding a detailed leukocyte formula. In particular, HAs employ a combination of flow cytometry and fluorescence utilising lysing buffers to achieve WBC differentials. Cells are classified based on their internal complexity and size which are analysed through “side” and “forward scatter” signals (SS, x-axis and FS, z-axis). In addition, fluorescent analysis (FL, y-axis) assesses the nucleic acid content into cells. Instruments such as Sysmex (XN-9000) and the more recently Mindray instrument (BC-6800 Plus Hematology Analyzer) are advanced multi-parametric HAs. These devices are particularly useful in COVID-19 cases for identifying atypical and pathological WBC scattergrams including neutrophilia, lymphopenia and a distinctive “sandglass” form (as reviewed in Figure 5).

Several studies, including those using instruments routinely employed in our laboratory, have identified a distinctive "sandglass effect" as the most significant feature for COVID diseases. This effect is characterized by the appearance of a discontinuous cluster of lymphocytes, represented by more than four dots in the upper part of the scattergram. Notably, this particular scattergram pattern has been reported in COVID-19 patients but not in those with other viral infections [76,77]. The identified lymphocyte cluster corresponds to a continuous cluster of large, hyperbasophilic (plasmacytoid) lymphocytes. Figure 5 illustrates the typical distribution of cell clusters on the white blood cell differential fluorescence (WDF) scattergram, generated by the Sysmex XN-9000 (A, C) and Mindray BC-6800 Plus (B, D) in COVID-19 patients observed in our laboratory. In both Sysmex and Mindray analysis, WBCs such as neutrophils (NE), lymphocytes (LY), monocytes (MO) that are differentiated using forward scattered light (FSC) to reflect cell size (NE-FSC, LY-Z, and MO-Z), side fluorescence light (SFL) to measure nucleic acid content (NE-SFL, LY-Y, and MO-Y), and side scattered light (SSC) to assess cell complexity (NE-SSC, LY-X, and MO-X). In COVID-19 patients, an increase in the fluorescent signal is particularly evident, with the NE-SFL parameter indicating heightened fluorescence in neutrophils (NE) and the HFLC (high fluorescence lymphocyte cells) parameter highlighting increased fluorescence in lymphocytes (LY) [78,79]. This heightened fluorescence is attributed to the presence of lymphoplasmacytoid cells and plasma cells, (both components product during the activation of the adaptive immune system), whose presence in blood indicates an immune response to infection [80]. Additionally, the LY-Y parameter, similar to the SFL for NE, reflects an increase in nucleic acid synthesis and cell activation in response to the infection, further correlating with COVID-19 severity [78]. The study by Urrechaga et al. highlights that COVID-19 patients also exhibit an increase in the NE-Y parameter when analyzed using the Mindray analyzer, which demonstrates high accuracy in predicting COVID-19 [81]. The WDF scattergram thus serves as an effective screening tool for managing suspected COVID-19 cases and predicting disease severity. Compared to the Sysmex analyzer, the Mindray BC-6800 offers a significant advantage with its three-dimensional (3D) graphical evaluation of cellular clusters. In fact, the 3D scattergram can be rotated, enabling observation of the cells from multiple angles, enhancing diagnostic accuracy [82]. Regarding microscopic resolution and the ability to use specific functions aimed at identifying other characteristics (such as abnormal lymphocytes or blasts), the DASIT instrument is highly detailed. However, in COVID-19 patients, both instruments, Mindray and Sysmex, are able to offer equal accuracy in the evaluation of scattergrams.

## Conclusions

The findings reviewed in this study are particularly valuable in describing and clarifying the biological alterations of blood cells induced by COVID-19 infection. In summary, the analysis of hematological parameters reported in several papers and reviewed here indicates that neutrophilia and lymphopenia are the primary characteristics in patients with severe forms of COVID-19 and in non-survivors, corresponding to an increased NLR. The significant decrease in lymphocytes is further confirmed by membrane immunophenotyping, which shows a marked reduction in the percentage of CD4 (helper) and CD8 (cytotoxic) lymphocytes, along with a significant increase in the CD4+/CD8+ ratio. Microscopic examination of blood cells reveals that the observed alterations in neutrophils, lymphocytes, monocytes, platelets, and red blood cells are closely linked to the progression of disease severity. In COVID-19, scattergram analysis reveals neutrophilia, and in many severe cases, a distinctive "sandglass pattern" in the positional distribution of lymphocytes (WDF) is associated with disease severity. In conclusion, this review highlights the alterations in blood cells due to COVID-19 infection, providing clinicians and researchers with valuable insights into the pathophysiological progression of the disease's severity. The review contributes to a better understanding of the hematological markers that could serve as key predictors of disease progression and poor outcomes.

## Appendices

Abbreviations

SARS-CoV-2: Severe Acute Respiratory Syndrome Coronavirus 2; COVID-19: Coronavirus Disease 19; M: Moderate; SE: Severe; S: Survivors; NS: Non survivors; CBC: Complete blood count; WBCs: White blood cells; RBCs: Red blood cells; ACE2: Angiotensin-converting enzyme 2; NK cells: Natural killer cells; NLR: Neutrophil-lymphocyte ratio; PLR: Platelet-lymphocyte ratio; ANC: Absolute neutrophil count; ALC: Absolute lymphocyte count; ABC: Absolute basophil count; AEC: Absolute eosinophil count; AMC: Absolute monocyte count; PLT: Platelets count; Hb: Hemoglobin; PBS: Peripheral blood smear; WDF: White blood cell differential fluorescence; FSC: Forward scattered light; SSC: Side scattered light; SFL: Side fluorescence light; NE-FSC: Forward-scattered light intensity of the neutrophils area on the WDF scattergram; LY-Z: Forward-scattered light intensity of the lymphocytes area on the WDF scattergram; MO-Z: Forward-scattered light intensity of the monocytes area on the WDF scattergram; NE-SFL: Fluorescent light intensity of the neutrophils area on the WDF scattergram; LY-Y: Fluorescent light intensity of the lymphocytes area on the WDF scattergram; MO-Y: Fluorescent light intensity of the monocytes area on the WDF scattergram; NE-SSC: Lateral scattered light intensity of the neutrophils area on the WDF scattergram; LY-X: Lateral scattered light intensity of the lymphocytes area on the WDF scattergram; MO-X: Lateral scattered light intensity of the monocytes area on the WDF scattergram; HFLC: Count of the upper lymphocytes area of the WDF scattergram.
